# Anthropogenic noise disrupts acoustic cues for recruitment

**DOI:** 10.1098/rspb.2024.0741

**Published:** 2024-07-24

**Authors:** Brittany R. Williams, Dominic McAfee, Sean D. Connell

**Affiliations:** ^1^ Southern Seas Ecology Laboratories, School of Biological Sciences, The University of Adelaide, Adelaide 5005, Australia; ^2^ Environment Institute, The University of Adelaide, Adelaide 5005, Australia

**Keywords:** acoustic enrichment, anthropogenic noise, ecosystem restoration, oyster, recruitment

## Abstract

Anthropogenic noise is rising and may interfere with natural acoustic cues used by organisms to recruit. Newly developed acoustic technology provides enriched settlement cues to boost recruitment of target organisms navigating to restoration sites, but can it boost recruitment in noise-polluted sites? To address this dilemma, we coupled replicated aquarium experiments with field experiments. Under controlled and replicated laboratory conditions, acoustic enrichment boosted recruitment by 2.57 times in the absence of anthropogenic noise, but yielded comparable recruitment in its presence (i.e. no boosting effect). Using the same technique, we then tested the replicability of these responses in real-world settings where independently replicated ‘sites’ are unfeasible owing to the inherent differences in soundscapes. Again, acoustic enrichment increased recruitment where anthropogenic noise was low (by 3.33 times), but had no effect at a site of noise pollution. Together, these coupled laboratory-to-field outcomes indicate that anthropogenic noise can mask the signal of acoustic enrichment. While noise pollution may reduce the effectiveness of acoustic enrichment, some of our reported observations suggest that anthropogenic noise *per se* might also provide an attractive cue for oyster larvae to recruit. These findings underscore the complexity of larval behavioural responses to acoustic stimuli during recruitment processes.

## 1. Introduction

The global nature of human activity is increasing anthropogenic noise in the world’s natural ecosystems. Noises from shipping, machinery and construction, for example, are pervasive and pose serious environmental change that affects both terrestrial and marine animals [1]. Marine organisms appear particularly vulnerable to the intensification of anthropogenic noise because they use sound for a range of activities, including to sense their surroundings, navigate, communicate, avoid predators, and find mates and food [2]. To detect, interpret and use underwater sound, marine animals have evolved specialized receptors (e.g. otoliths and statocyst organs). Where other cues are spatially limited underwater (i.e. sight and olfactory cues), sound is the sensory cue that travels fastest and farthest independently of currents [3–5]. Therefore, the hearing ranges of marine animals dictate their response and potential vulnerability to anthropogenic noise [2]. Of concern, the duration, broad frequency range and intensity of marine anthropogenic noise now dominate a large part of the soundscape that was once filled with the biophony (the deliberate and incidental sound that arises from a biological source) of marine animals [2,6]. Studies have shown that locations with anthropogenic noise sources (e.g. large ships) affect low-frequency acoustic complexity, masking the natural variability of soundscapes at lower frequencies [7]. Shipping, motorboating, sonar and seismic exploration may potentially mask the biological signals that marine animals use for conspecific communication, navigation and recruitment. This is because they overlap with the hearing and communication ranges of many animals [8,9]. Such noises are often detrimental to marine animals, inducing physiological and behavioural changes [10–12], as well as stress, injury and mortality [2,13,14].

Anthropogenic noises also affect marine invertebrates, inducing physiological changes and organ damage, as well as influencing behaviour and communication [15–18]. For example, boat noise disrupts the recruitment processes of coral and barnacle larvae [19,20] and the orientation of juvenile crabs [21]. In extreme cases, the noise of seismic air guns, for example, can lead to developmental abnormalities in bivalve larvae [22]. On the other hand, mussel larvae can exhibit enhanced recruitment in the presence of vessel noise [23,24]. Despite these documented impacts, large knowledge gaps remain on how anthropogenic noise affects invertebrate larvae, especially during recruitment processes [25]. Recruitment is the process by which dispersing larval stages (e.g. meroplankton [26]) of a species settle into a benthic habitat (i.e. ‘settlement’) and transition to a more sedentary life stage [27]. Throughout this article, we use the term ‘recruitment’ to mean the measured input of juveniles to the benthic population, which is not necessarily the same as the number of larvae settling. To date, studies have indicated that anthropogenic noise may cause auditory masking of the natural sound cues used by recruiting animals, such as coral larvae [19], though most of this research has focused on fish and mammals [28]. Of note, there is little known on how anthropogenic noise may impact the recruitment of bivalve larvae of conservation importance, such as the larvae of reef-building oysters that use natural sound cues for navigation [29–31]. This is important because oyster reef restoration efforts are increasing worldwide and often occur along urbanized coastlines where noise from shipping, machinery and construction is intensifying [32,33]. This overlap is concerning because the anthropogenic noise arising from these activities potentially conflicts with the natural soundscapes that animals (e.g. bivalves) rely upon during settlement [2].

Acoustic enrichment of degraded biogenic sounds using underwater speakers may provide a novel tool for enhancing recruitment processes that improve conservation outcomes [34,35]. Underwater speakers can periodically replace the lost acoustic cues that diverse animal groups use to navigate towards and recruit into suitable habitats [36]. For example, playback of healthy habitat sounds can guide oyster larvae to sites targeted for restoration [29,30,35,37]. The exact mechanism by which they do this remains largely unknown. However, it seems likely that their statocyst organs (used for orientation relative to gravity) are used to detect gradients in particle motion generated by sound waves, as documented in other marine invertebrates [38–40]. Acoustic enrichment might offer a scalable, affordable solution for ecosystem restoration by enhancing a key component of successful restoration—organismal recruitment.

However, while we know that oyster larvae can respond to healthy reef sounds, the restorative value of acoustic enrichment in the presence of anthropogenic noise is currently unknown. Anthropogenic noise could potentially mask the signal produced by acoustic enrichment and disrupt recruitment processes. Alternatively, anthropogenic noise may lie within the desirable range of sound frequencies that attract larvae. It is important to understand this nuance and whether acoustic enrichment can yield positive outcomes in places with anthropogenic noise, especially considering that many restorations occur along urbanized coastlines [37] characterized by anthropogenic noise. In this study, we focus on the larvae of Australia’s flat oyster (*Ostrea angasi*) that is being restored from functional extinction. We assess the potential context dependency by which acoustic enrichment can overcome a major challenge to ecosystem restoration; achieving sufficient natural recruitment of oyster larvae to restore reefs situated in noisy places. We coupled aquarium with field experiments to determine the value of using acoustic ‘enrichment’ for oyster reef restoration in localities associated with anthropogenic noise. In the aquarium, we tested whether acoustic enrichment, with or without anthropogenic noise, could boost the recruitment of oysters relative to ambient controls. In the field, we tested the effectiveness of acoustic enrichment on the natural recruitment of larval oysters across two restoration sites: one associated with a relatively quiet, natural soundscape and one where anthropogenic noise was present. The importance of coupling these laboratory-to-field outcomes centres on replicability; being able to replicate similar responses through controlled laboratory experiments and extend their replicability to real-world settings where independently replicated sites are not feasible.

## 2. Methods

### (a) Reef soundscape playback recordings

For the acoustic enrichment playback recordings in both the aquarium and field experiments, we used soundscape recordings captured from a local natural rocky reef in Gulf St Vincent, South Australia (Port Noarlunga Reef). Flat oyster reefs covered thousands of kilometres of the Australian coastline 200 years ago, including Gulf St Vincent, but today no flat oyster reefs remain on mainland Australia [41]. Therefore, we prioritized sampling the healthiest reef soundscape available in the gulf (determined from sound pressure levels and snapping shrimp snaps identified by sampling multiple sites and habitat types in the gulf) [36]. Because the intent of this study was to enrich acoustic cues that would enhance larval recruitment, and was not to reproduce real-world conditions (i.e. daily cycles in biophony), we used soundscape recordings made within 1 h of sunrise (i.e. the ‘dawn chorus’) that are characterized by loud snapping shrimp crackle and demonstrated to enhance recruitment [36]. This dawn soundscape is the most biologically active time of the day for our local reefs [36,42] and across other reef systems [29,30,43,44]. Soundscape recordings were made during the recruitment season for the flat oyster (austral summer, December), at high tide (4–8 m of water) to minimize the contribution of other sound sources (i.e. wind and breaking waves) to our recordings. We took recordings continuously for an hour, using four calibrated Sound Trap 202 hydrophones (Ocean Instruments (Auckland, New Zealand), frequency response 0.1–30 kHz, set to high gain sensitivity (−169 to −169.8 dB re 1 V μPa^–1^), −3 dB bandwidth of 21.6 kHz, 48 kHz sampling frequency, data digitized using a 16 bit resolution). Hydrophones were anchored 1 m above the seafloor using a subsurface buoy to suspend them. For the playback experiments, we created a looped 1 min long sound file in the program Audacity. This consisted of four random 15 s long sound snippets (one from each hydrophone), which were in sequence and non-overlapping. We then analysed the spectral characteristics of this recording (for details see electronic supplementary material, Appendix S1, §S2).

### (b) Aquarium experiments

We performed our aquarium experiments at the University of Adelaide aquarium using hatchery-reared pediveliger oyster larvae (*O. angasi*; 6–10 days old, consistent across trials) supplied by the South Australian Research and Development Institute hatchery, where they were fed daily. Larvae were used within 2 days of arrival and were maintained before experiments in aerated aquarium tanks without food, during which time they displayed swimming behaviour and an actively searching foot that enabled them to move and settle. This species is a brooding oyster that releases one to three million veliger larvae (170–189 µm) [45] during months where mean seawater temperatures exceed 17°C (austral summer) [46]. These larvae spend several days to 2 weeks in the water column, potentially dispersing tens of kilometres [47], after which they can explore the seafloor before permanently attaching to the substrate as ‘spat’.

To test the effectiveness of acoustic enrichment for oyster recruitment in the presence of anthropogenic noise, we exposed oysters to four sound treatments: (i) a healthy reef soundscape (‘Reef’), (ii) a healthy reef soundscape in the presence of anthropogenic noise (‘Reef + Noise’), (iii) a soundscape filled with anthropogenic noise (‘Noise’) and (iv) a no sound control (‘Control’). Our healthy reef soundscape was recorded from Noarlunga Reef (described above). For the anthropogenic noise recording, we used a combination of sounds considered to be the most ubiquitous anthropogenic noises in the marine environment: shipping, motorboating, pile-driving and near-shore urban noises [2]. Each of these sound recordings came from ‘Freesound’, a collaborative repository of creative commons licensed audio samples. The ‘Freesound’ recordings we chose were compared among a variety of available sound files for each sound type. They were selected by considering factors such as sound pressure level and frequency characteristics, aiming to capture the nuances of the anthropogenic noises typically described in the literature as widespread in coastal ecosystems [31]. Each recording had been recorded from the field using hydrophones, but specific details on the depth and location of the recordings were not provided. In the program Audacity, we overlaid each of these 1 min long recordings to form a combined, looped 1 min long sound file. These particular sources of anthropogenic noise are all increasing along urbanized estuaries and coastlines [1], which may be a concern for marine restorations focused on modified estuarine environments. While our field sites (described in §2c) do not contain frequent pile-driving activity, we included this noise source in our aquarium experiments because pile-driving is a recognized environmental issue owing to the high noise levels produced during coastal marine construction (i.e. construction of bridges, wharves and mariners) [48,49]. In addition, it is important to note that our aquarium experiments were not representative of real-world soundscape conditions and did not replicate the soundscapes in our field experiments. Rather than replicate these, our intention was to test whether anthropogenic noise disrupts sounds used by larvae in a controlled environment, and therefore might limit the effectiveness of acoustic enrichment upon recruitment, a technique that is shown to attract oyster larvae [29–31,35].

We played our recordings using underwater speakers that we constructed with our technology collaborators (Australian Ocean Lab) using off-the-shelf materials (5 × 3 cm vibration loudspeaker (25W, 4 Ω, omnidirectional sound, frequency response 0.3–20 kHz; unbranded), an audio amplifier (MAX9744 amplifier; Adafruit Industries, New York City, USA), a 64 bit processor (Raspberry Pi 3 Model B+) and one rechargeable battery for power (12V SLA; RS Components Pty Ltd, London, UK), secured inside waterproof PVC housing; H × W: 10 × 12 cm; (https://www.ausocean.org/technology). For the ‘Reef + Noise’ treatment, we used two speakers: one to play the reef recording, and one to play anthropogenic noise. For the ‘Control’, ‘Reef’ and ‘Noise’ treatments, we used a single speaker, accompanied by an additional dummy speaker to ensure an equivalent number of speakers in every treatment to account for confounding effects of the speaker structure. We played all recordings at the highest volume on the amplifier and parameterized the sound treatments (for details see electronic supplementary material, Appendix S1, §S1).

Our oyster larvae remained in peak condition (i.e. actively searching foot) for the 2 day period they were in the aquarium. Over this time, we ran three trials (total *n* = 3 per treatment) where we exposed larvae to our sound treatments by randomly assigning and placing the speakers into four plastic tanks filled with seawater (9 l; L × H W: 31 × 22 × 18 cm; 20°C). To limit background noise, these tanks were soundproofed using acoustic foam (5 mm thick self-adhesive sound-absorbing foam, Jaycar). To generate the darker conditions that encourage oysters to settle, we covered the tanks iwithcloth (Grunt black builder’s film [50,51]. Within each tank, we placed three 70 ml plastic specimen jars filled with seawater and containing a tile (2.5 × 2.5 cm sanded PVC square) for larvae to use as a substrate, and approximately 300 pediveliger oyster larvae (18 ml pipette of larvae at approximately 16.67 oysters ml^−1^) that were exposed to sound treatments for 2 h. In contrast to our month-long field experiment observing wild recruitment, our aquarium study was restricted to a shorter duration because the pediveliger larvae were ready to recruit, and this was sufficient time for a proportion of the larvae to react to the treatments (pilot studies of longer duration resulted in almost all larvae recruiting, obscuring treatment effects). After each trial, we removed the specimen jars and counted the number of oysters that had been recruited onto each tile using a dissecting microscope. We gently agitated the larvae using water from a pipette to ensure proper attachment and discounted any crushed larvae. Owing to the small size of these specimen jars, we were unable to place our hydrophones directly inside the specimen jars to record the *in situ* soundscape, but instead placed hydrophones within each tank to demonstrate differences among treatments, as other studies have done [29,30]. To ensure there were no experimental artefacts from individual tanks or speakers, between each experimental run we repositioned the sound treatments in the aquarium and switched the speakers between sound treatments.

To analyse our data, we first calculated the variation between tiles within a tank for each treatment within each trial, using the coefficient of variation. In each of these cases, the coefficient of variation was lower than 1, indicating low variance between tiles within each tank. Therefore, we calculated the mean recruitment across the three tiles within each tank to provide a solitary value per tank, per trial (*n* = 3 replicates per treatment). We used these three values to calculate the mean recruitment and standard error per treatment across all trials. We performed one-way ANOVAs to assess any significant differences in recruitment between treatments, ensuring the model assumptions were met. First, we compared the ‘Reef’ and ‘Control’ treatments, which signified natural reef conditions. Second, we compared the ‘Reef + Noise’ and ‘Noise’ treatments, which signified anthropogenic conditions. We also standardized these recruitment values to ‘mean recruitment per 15 cm^2^’ to enable comparison between the larger panels used in our field experiments (see below). Finally, we calculated the effect size and standard errors of boosted recruitment between the ‘Reef’ and ‘Control’ treatments, and then the ‘Reef + Noise’ and ‘Noise’ treatments, using the standardized mean difference (Cohen’s *d*) and the bootstrap procedure [52]. All analyses were performed in R (v.4.1.2).

### (c) Field experiments

We conducted our field experiments at two flat oyster restoration sites in Gulf St Vincent, South Australia. This gulf was once characterized by vast flat oyster reefs, but this ecosystem has since been lost to an oyster dredging fishery in the late nineteenth century [53] and replaced by relatively barren sand flats [54]. Flat oysters are now the focus of a nationwide reef restoration programme in Australia [55]. Our field sites included two oyster restorations with different ambient soundscapes: (i) ‘Natural Soundscape’ (Glenelg Reef, 34˚58.38′ S, 138°29.88′ E), a relatively quiet, natural soundscape characterized by low-intensity snapping shrimp snaps and periodic boating activity during the day, is located approximately 1 km off Adelaide’s metropolitan coastline and was constructed in November 2020, and (ii) ‘Anthropogenic Soundscape’ (Port Adelaide Reef, 34˚50.70′ S, 138°29.88′ E), an urbanized soundscape characterized by frequent shipping, boating and relatively constant traffic noises, and is the site of a 2018 restoration in a heavily urbanized waterway. Each of these restorations is located in 4–8 m of water where natural recruitment of native oysters has been observed in high numbers in cases where sufficient substrate has been provided for them [35]. Our experiments were performed throughout the oyster recruitment season (i.e. October 2020 to March 2021 [46]).

We observed the rates of wild *O. angasi* larval recruitment across these two sites when exposed to ambient conditions (‘Control’) and with playback of our looped reef soundscape (‘Enriched’). Here, we define the term ‘acoustic enrichment’ as the full level of sound produced by our marine speakers, and the term ‘masking of soundscapes’ as the amount of interference that anthropogenic noise has upon acoustic enrichment. We quantify this as the difference in sound pressure levels at a site between sound playback (i.e. acoustic enrichment) and the ambient soundscape (i.e. a natural ambient soundscape or a soundscape containing anthropogenic noise), where a smaller difference indicates greater soundscape masking. In the ‘Enriched’ treatment, we used underwater speakers to play the reef soundscape (larger version of the speaker described above, including four rechargeable batteries for power, secured inside waterproof PVC housing; H × W: 10 × 12 cm). In the ‘Control’ treatment, we used dummy control speakers that consisted of the waterproof PVC housing without the encased electronics. A better control might be a speaker without noise but with electronics in place and active as there may be a confounding effect of an electrical field [56]. Any potential discovery of elevated recruitment from electrical fields would represent a substantive discovery *per se*.

**Figure 1.**
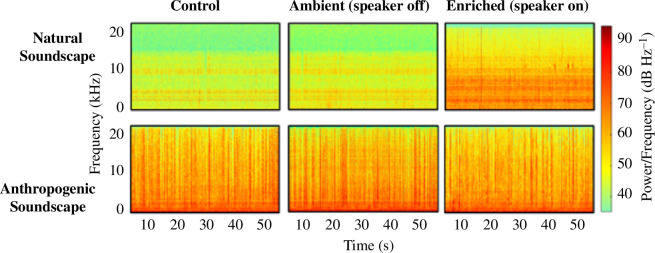
Spectrograms in the field for the ‘Control’ and ‘Enriched’ treatments, and the background ambient soundscape (60 s long recordings) across two restoration sites; ‘Natural Soundscape’ and ‘Anthropogenic Soundscape’. Spectrograms were produced using 1 s windows with 50% overlap.

The natural reef recording we played was the same as described in our aquarium experiments. To parameterize our playback treatments at each site, we deployed hydrophones to record the soundscapes of each treatment at each of the reef locations (figure 1; for more details see electronic supplementary material, Appendix S1, §S2). For each site, these recordings were made on one single day at the beginning of the experiment, and therefore we did not capture daily variability in these soundscapes. Despite this, previous soundscape recordings made at these sites (Connell, unpublished data from the sites surveyed by authors, 2020-21) demonstrate that the ‘Natural Soundscape’ (Glenelg Reef) is characterized by low-intensity snapping shrimp snaps that peak within an hour of sunrise, and periodic boating activity during the day. Meanwhile, ‘Anthropogenic Soundscape’ (Port Adelaide Reef) is characterized by frequent shipping and boating noise, as well as relatively constant traffic noises, as a result of it being a highly urbanized coastline. At the ‘Natural Soundscape’ reef, the ‘Enriched’ treatments were shown to significantly increase sound pressure levels and snapping shrimp snap counts relative to ‘Control’ (figure 1; 8.90 dB Hz^–1^ increase, 435 snaps min^−1^ increase). By contrast, the ‘Enriched’ treatment at the ‘Anthropogenic Soundscape’ reef did not significantly enrich sound levels or snapping shrimp snaps relative to ‘Control’. The ambient soundscape at this site did contain a larger number of snapping shrimp snaps compared with ‘Natural Soundscape’, possibly due to it being an older restoration; however, it was also associated with frequent shipping, boating and urban noises (0.01–10 kHz). Finally, our self-constructed speakers could only partially recreate the acoustic characteristics of our natural reef recording (for more details see electronic supplementary material, Appendix S1, §S2), but was still able to enrich the ambient soundscape at the sites associated with natural sounds (figure 1).

For the ‘Natural Soundscape’, we had two replicate sites per ‘Control’ and ‘Enriched’ treatment and performed experiments across three trials (*n* = 6). For the ‘Anthropogenic Soundscape’, we had two replicate sites per treatment and performed experiments across two trials (*n* = 4). At each of the two locations, each speaker and dummy control were separated by at least 50 m to avoid sound crossover between treatments, and secured 0.5 m above the seafloor. To provide a substrate for larvae to recruit to, we secured a vertically oriented panel (15 × 15 cm; concrete board in which larval oysters can settle [35]) to the top of a plastic stake, which we inserted 0.3 m a.s.l. We placed six of these stakes (four for ‘Anthropogenic Soundscape’) 2 m away from each speaker or dummy control, with each stake spaced at least 1 m apart from one another. Our speakers played the sound recording loop continuously for a month, as the early stages of reef development are crucial to the success of oyster reef restorations [35]. At the end of each trial, we removed the panels and counted the number of oysters recruited to the outward-facing side of the panel under a dissection microscope.

We calculated the mean recruitment and standard errors per treatment across each of the two restoration sites, and then tested for significant differences between treatments using the Welch *t*‐test, after ensuring the data met the assumptions for this test. Furthermore, we calculated the effect size means and standard errors of recruitment and of boosted root-mean-square sound pressure levels (SPL_rms_) between treatments. We did this using the standardized mean difference (Cohen’s *d*) and the bootstrap procedure. All analyses were performed in R (v.4.1.2).

## 3. Results

### (a) Aquarium experiments

**Figure 2.**
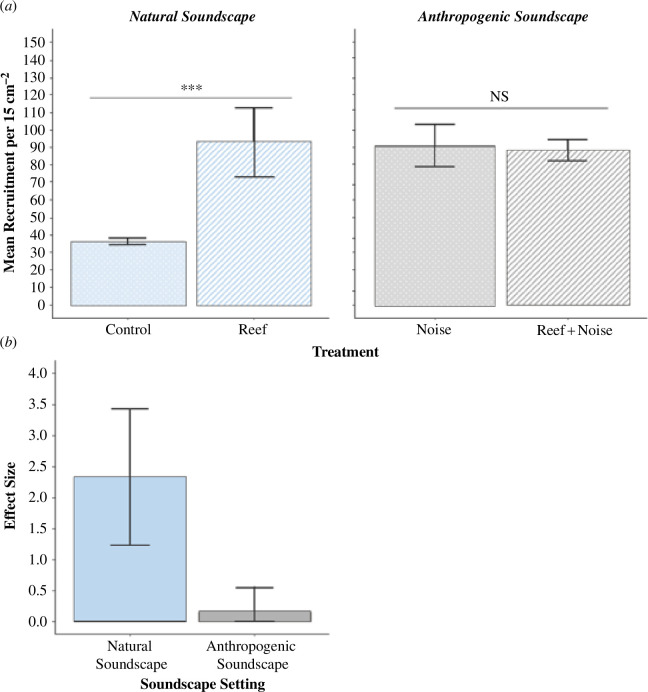
(*a*) Mean (±1 s.e.) oyster recruitment in the aquarium between (left) ‘Control’ and ‘Reef’ treatments in a natural soundscape setting and between (right) ‘Noise’ and ‘Reef + Noise’ treatments in an anthropogenic soundscape setting (*n* = 3). Significant (***) and non-significant (‘NS’) differences between treatments are denoted on lines above columns. (*b*) Effect sizes on larval recruitment (Cohen’s *d* ±1 s.e.) from acoustic enrichment between the ‘Control’ and ‘Reef’ treatments (‘Natural Soundscape’) and the ‘Noise’ and ‘Reef + Noise’ treatments (‘Anthropogenic Soundscape’).

Analysis of the aquarium experiments revealed a significant effect of acoustic enrichment upon recruitment relative to controls in conditions associated with natural sounds (one-way ANOVA; *F*_1,4_ = 8.158, *p* = 0.046; figure 2*a*). The ‘Reef’ sound treatment significantly increased the density of larvae by 2.57 times relative to the ‘Control’ treatment (mean recruitment 15 cm^2^ (±1 s.e.): 92.64 ± 34.20 and 36.00 ± 3.48, respectively). By contrast, the ‘Reef + Noise’ treatment received no significant increase in the density of larvae relative to the ‘Noise’ treatment (mean recruitment 15 cm^2^ (±1 s.e.): 88.02 ± 10.56 and 90.66 ± 21.18, respectively; figure 2*a*). Furthermore, the effect size of boosted recruitment between ‘Reef’ and ‘Control’ treatments in conditions associated with natural soundscapes (Cohen’s *d* (±1 s.e.), 2.33 ± 1.10) was 14.56 times greater than that between ‘Reef + Noise’ and ‘Noise’ treatments in conditions associated with anthropogenic soundscapes (Cohen’s *d* (±1 s.e.), 0.16 ± 0.39) (figure 2*b*). The findings from these controlled aquarium experiments lay the foundation for testing the mediating influence of anthropogenic noise on the facilitative process of acoustic enrichment on recruitment. The key advancement would be the replicability of these experimental outcomes in real-world settings, where independently replicated sites are not always feasible.

### (b) Field experiments

#### (i) Effect sizes of recruitment and soundscape characteristics

**Figure 3.**
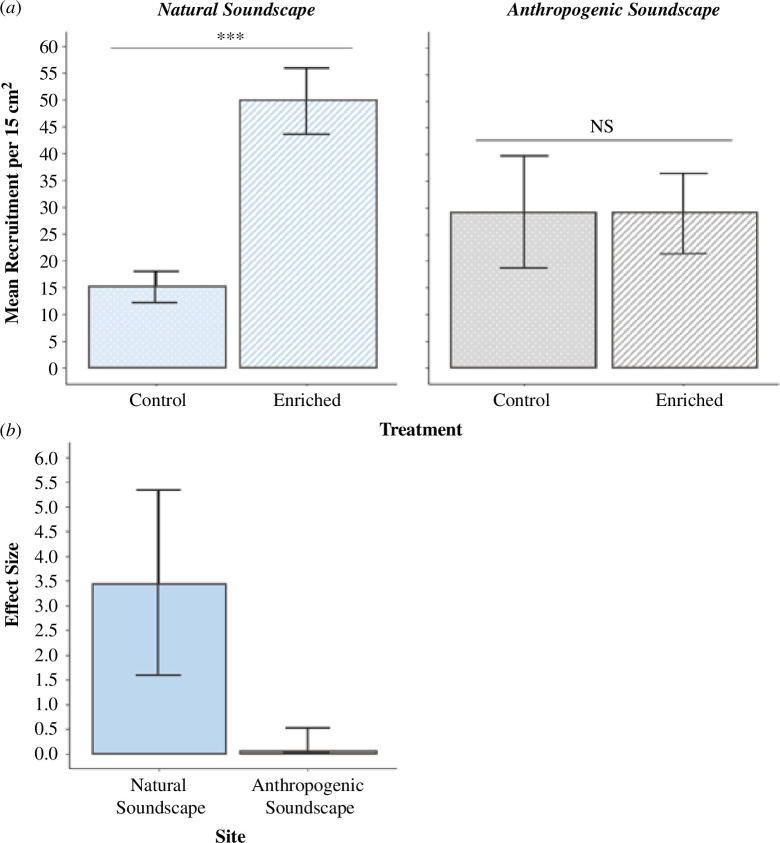
(*a*) Mean (±1 s.e.) oyster recruitment in the field between ‘Control’ and ‘Enriched’ treatments across two restoration sites; ‘Natural Soundscape’ (*n* = 6 per treatment) and ‘Anthropogenic Soundscape’ (*n* = 4 per treatment). Significant (***) and non-significant (‘NS’) differences between treatments are denoted on lines above columns. (*b*) Effect sizes on larval recruitment (Cohen’s *d* ±1 s.e.) from acoustic enrichment at the relatively quiet (‘Natural Soundscape’) and noise-polluted (‘Anthropogenic Soundscape’) oyster restoration sites.

Analysis of recruitment in the field revealed higher recruitment in ‘Enriched’ treatments than ‘Control’ treatments at the ‘Natural Soundscape’ site, but not at the ‘Anthropogenic Soundscape’ site (figure 3*a*). At the ‘Natural Soundscape’ site, oyster recruitment to the ‘Enriched’ treatment (49.89 ± 6.08 oysters 15 cm^2^) was significantly increased by 3.33 times (*t*‐test: *t*(equation 49.90) = −5.19; *p* = 0.001) relative to the ‘Control’ treatment (15.02 ± 2.87 oysters 15 cm^2^). Whereas at the ‘Anthropogenic Soundscape’ site, no significant difference was detected in recruitment between ‘Enriched’ (28.90 ± 7.53 oysters 15 cm^2^) and ‘Control’ treatments (29.20 ± 10.53 oysters 15 cm^2^) (*t*‐test: *t*(equation 27.16) = 0.02; *p*>0.05; figure 3*a*). In terms of the effect of acoustic enrichment at each site, the boost to recruitment between the ‘Control’ and ‘Enriched’ treatments was 138.08 times greater at the ‘Natural Soundscape’ site (Cohen’s *d* (±1 s.e.), 3.452 ± 1.89) compared with the ‘Anthropogenic Soundscape’ site (Cohen’s *d* (±1 s.e.), 0.025 ± 0.49) (figure 3*b*). These field outcomes are aligned with the laboratory outcomes, which are needed to meet the challenge of replicability in real-world anthropogenic soundscapes.

#### (ii) Soundscape characteristics

**Figure 4.**
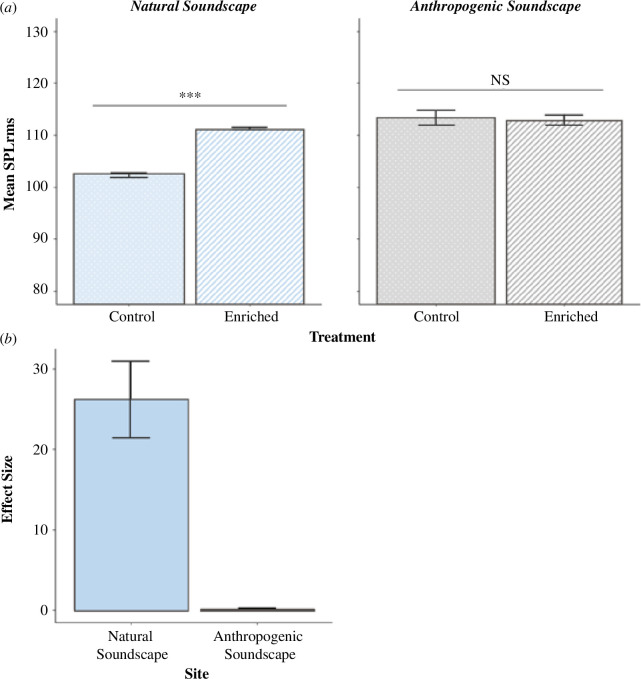
(*a*) Mean (±1 s.e.) sound pressure level (SPL_rms_) in the field between ‘Control’ and acoustically ‘Enriched’ treatments across two restoration sites; ‘Natural Soundscape’ and ‘Anthropogenic Soundscape’ (*n* = 4). Significant (***) and non-significant (‘NS’) differences between treatments are denoted on lines above columns. (*b*) Effect sizes (Cohen’s *d* ± 1 s.e.) of boosted sound (derived from SPL_rms_) between the ‘Control’ and acoustically ‘Enriched’ treatments across our two restoration sites.

At the ‘Natural Soundscape’ site, there was a significant difference in mean sound levels (SPL_rms_) (figure 4*a*) and mean snaps min^–1^ (snaps) between ‘Enriched’ (mean snaps (±1 s.e.), 547 ± 40.90) and ‘Control’ treatments (mean snaps (±1 s.e.), 112 ± 4.43) (SPL_rms_ *t*-test: *t*(6) = 45.13; *p* = 0.001; snaps *t*‐test: *t*(6) = 9.88; *p* = 0.002) (electronic supplementary material, Appendix S1, §S2). At the ‘Anthropogenic Soundscape’ site, the mean SPL_rms_ and mean snaps were statistically indistinguishable (figure 4*a*) between ‘Enriched’ (mean snaps (±1 s.e.), 405.50 ± 3.60) and ‘Control’ treatments (mean snaps (±1 s.e.), 353.80 ± 42.40) (electronic supplementary material, Appendix S1, §S2). At the ‘Natural Soundscape’ site, the effect size of boosted sound (derived from SPL_rms_) between ‘Control’ and ‘Enriched’ treatments (Cohen’s *d* (±1 s.e.), 26.180 ± 4.73) was 128.97 times greater than that at the ‘Anthropogenic Soundscape’ site (Cohen’s *d* (±1 s.e.), 0.203 ± 0.05) (figure 4*b*).

## 4. Discussion

Our study supports the known benefits of acoustic enrichment [29,30,34,35], yet we identify an important context dependency; the potential failure of acoustic enrichment to boost recruitment under anthropogenic noise. Furthermore, our study indicates that anthropogenic noise might still lie within the range of sound frequencies that can induce oyster larvae to settle. To provide tests that assessed this context dependency, we explicitly tested for the replicability of results between the laboratory and field. The near impossibility of finding independent and comparable replicates of anthropogenic sound in the field is likely to reflect many other coastal settings. This is due to the complex and dynamic nature of coastal environments, where numerous factors contribute to the variability in anthropogenic noise (e.g. different sound sources and intensities and local geomorphology) [57,58]. Valuable future research could focus on using gradients of anthropogenic noise inherent among the world’s urbanized coastlines.

In each test in the aquarium and field, we show that we can boost oyster recruitment by two to three times using acoustic enrichment in the absence of anthropogenic noise. However, in each of these settings, acoustic enrichment had no boosting effect when in the presence of anthropogenic noise. Anthropogenic noise is known to impact fish and mammals in a variety of ways [2,28] and our study suggests that it might also influence the recruitment of invertebrate larvae by overlapping with the acoustic signals larvae use for navigation. For example, the comparable larval recruitment between treatments in the anthropogenic noise setting suggests that this noise overlaps with the range of frequencies to which oyster larvae respond, thus complicating the discrimination between the functional use of natural and anthropogenic noise by larvae. Regardless, this study indicates that anthropogenic noise masks acoustic enrichment, potentially rendering this technique ineffective along noisy coastlines.

### (a) Acoustic enrichment and recruitment in an Anthropocene ocean

The recruitment process works to replenish populations and is intricately linked to the functioning and maintenance of a healthy ecosystem [59], especially where reef-building foundation species are concerned (e.g. oysters and coral). Successful recruitment requires larvae to distribute in the water column and select an appropriate habitat by responding to visual, chemical and acoustic stimuli [60–62]. However, human activities can modify these cues, such as the generation of artificial light at night [63] and alterations to the soundscape from human activities. Oyster reef restorations are increasingly occurring in metropolitan estuaries and coastlines [37], with these soundscapes being dominated by anthropogenic noise that might disrupt recruitment processes. This is because anthropogenic noise might overlap with the frequencies of biological sounds that species targeted for restoration rely upon to find suitable adult habitat [64]. However, it might also damage the sensory organs with which larvae navigate [65]. Building upon previous work that demonstrates acoustic enrichment works to boost larval oyster recruitment [31,35,36], this study found that acoustic enrichment does not provide this boosting effect in the presence of high anthropogenic noise.

In the aquarium, we demonstrate that acoustic enrichment can increase recruitment when anthropogenic noise is absent, relative to a no sound control that represents a quiet site. However, acoustic enrichment provides no significant boost in recruitment when in the presence of noise (relative to a noise treatment that represents a noisy site). Surprisingly, comparable recruitment was observed between the anthropogenic noise treatments and the healthy reef treatment. This indicates that larvae might not distinguish between these sound types [66,67] or that anthropogenic noise can still induce high recruitment rates; a phenomenon that has also been observed with mussels [23,24]. While we observed similar recruitment in the aquarium, both sound treatments induced settlement in a confined space (i.e. the specimen jar); therefore, it remains unclear whether anthropogenic noise can provide similar directionality that natural soundscapes provide to guide recruiting organisms [31]. A key avenue for future research would be to determine if larvae are attracted to anthropogenic noise and can interpret its direction, or whether it provides no directional cue. If the latter is true and anthropogenic noise disorientates larvae, resulting in more time spent in the water column, it could impact their energy expenditure with ramifications for their post-recruitment survival [68]. Likewise, if larvae are attracted to anthropogenic noise for navigation and settlement, then acoustic enrichment may not be an appropriate technique for oyster restoration in such places. Meanwhile, our field experiments demonstrate that acoustic enrichment can boost recruitment at relatively quiet sites with some snapping shrimp snaps, but not at noisy sites with frequent shipping, motorboat and urban noises. The lower recruitment observed at the ‘Anthropogenic Soundscape’ site may have been due to a loss of directionality of the playback recording as a result of the anthropogenic noise dominating the soundscape. However, testing this across other sites with anthropogenic noise would offer a valuable comparison to better determine the causes of this effect and any context dependency. Overall, our findings suggest that acoustic enrichment may only provide an effective navigational cue where there is little anthropogenic noise.

The comparability of controlled and replicated laboratory experiments with controlled and unreplicated field experiments was treated as a unit of interpretation. The result was reproducible across two different research domains. Each domain has its own inherent weakness. Laboratory results are limited by laboratory artefacts that do not reflect the true nature of the phenomenon under investigation. Field experiments are limited by inherent spatial variation of an infinite number of variables that can never be controlled and their measure only highlights natural spatial variation. The strength of our results is that in spite of these challenges, the result could be reproduced independently between the domains.

Both volume and type of sound may affect larval behaviour. An increase in sound level, anthropogenic or natural, might increase recruitment up to a point, and each of these ‘types of sound’ appears to drive differences in recruitment. We found tentative evidence for elevated recruitment in the presence of anthropogenic noise in the field (see §2(b)), which had the additional effect of masking the effect of acoustic enrichment. So, future work might aim to illuminate whether larvae can detect and respond to specific sounds, through analysing responses to each frequency, sound pressure level and importantly, particle motion level, which is largely understudied [69]. This would enable us to identify the sounds that are attractive and unattractive to target organisms. For example, statocysts likely enable detection of particle motion and are described for the pediveliger stages of some bivalve species [70–72], including the European flat oyster [73]. With greater understanding of the sound detection mechanisms that enable larvae to detect and distinguish between sounds, we might reduce the uncertainty around observed variation in the attractiveness of sound among different species and the wide variety of environments in which they occur.

Furthermore, these results do not distinguish between other mechanisms that may have influenced the observed recruitment. While our previous work shows that larvae can actively move towards attractive sounds in the aquarium [31], the main disperser of larvae in the wild is transport via currents and tides. Our observed differences in recruitment might therefore have largely been dictated by currents carrying larvae across our sites, where they were then induced to settle, rather than larvae detecting attractive sound from afar and actively swimming towards it. In addition, an experimental consideration that this study did not address is the potential for the speaker electronics to generate low-frequency electromagnetic fields that are shown to impact larval development [56], and could possibly have influenced larval recruitment behaviour. Finally, there is a possibility that environmental sound may interact with, or be overwhelmed by, other anthropogenic (e.g. light) or environmental factors (e.g. salinity, oxygen, temperature and predators) that influence larval recruitment. Oysters use various cues across different spatial scales to navigate towards suitable recruitment sites, while environmental conditions such as water temperature dictate recruitment timing [46]. While water temperatures and salinity do not differ considerably between our two field sites, other cues that we did not record could have contributed to shaping the recruitment patterns we observed. For instance, in the anthropogenic setting, it is worth noting that there may be a higher presence of artificial light at night, which could have affected the oyster’s response to light cues. Furthermore, sites with low turbidity, high dissolved oxygen and low predation are shown to yield higher oyster recruitment [74]. While these environmental variables were not recorded in this study, the demonstrated boost to recruitment within our natural soundscape site emphasizes that acoustic stimuli exerts a strong influence on larval recruitment processes. Nevertheless, the impacts of artificial light and other environmental variables warrants further investigation in the context of our research, which might have influenced the difference in recruitment among sites observed in our study. As such research is replicated across the globe among its infinitely varying contexts, the reproducibility and context dependency of recruitment responses to anthropogenic noise will become increasingly understood as such replication has afforded other emergent disciplines (e.g. Connell and Leung [75]).

### (b) Implications for restoration practice

Acoustic enrichment guides oyster larvae navigation in the aquarium and field [31,35], yet anthropogenic noise may limit its effective application for oyster reef restorations. By overlapping with the signals of habitat-related sounds that larvae use to navigate, anthropogenic noise appears to limit the effectiveness of acoustic enrichment. This work adds to a growing body of marine and terrestrial research, which shows the implications that noise has for recruitment dynamics [76]. For example, in choice experiments, boat noise in the presence of reef sounds cause coral reef fish larvae to move away from the sound, contrasting their attraction response in the presence of reef sounds alone [77].

Importantly, the use of acoustic enrichment for enhancing restoration outcomes, as in this article, is intended for use in the first couple of months of a restoration, as shown in other studies [35]. It is not suggested as a long-term solution but rather a short-term restoration technique to be used alongside other techniques. It is important to note that playing a continuous sound like a dawn chorus long-term may be an issue for an ecosystem. This is because organisms use natural rhythms and temporal variations for timing and chronobiological decision-making and orientation [78]. They may also need quieter windows to channel their own communication needs [79]. It is therefore important that future studies consider a holistic ecosystem approach when considering acoustic enrichment to ensure it does not have adverse impacts upon other organisms.

The anthropogenic noises within urbanized waterways are known to dominate soundscapes [2]. For example, shallow waterways, such as the urbanized site in this study, generally have higher noise pollution owing to the reflection of sound waves from the bottom and sides of the waterway [80], with this reflection enhanced if the waterway is constructed with dense concrete material [81]. In such urban waterways, the concrete walls may cause sound to bounce unpredictably in various directions, leading to a loss of acoustic directionality. As we observe an increasing trend of restoration efforts in noisy, metropolitan waterways, it is important to consider the potential limitations of acoustic enrichment. This potential loss of acoustic directionality due to concrete structures might disperse sound waves in ways that may not effectively serve the intended purpose of acoustic enrichment, further emphasizing the challenges in mitigating anthropogenic noise in such environments. Where sites are characterized by high levels of noise pollution, they may be inappropriate for restoration and the use of acoustic enrichment to enhance recruitment of the target species. Likewise, if noisy sites can still yield comparable recruitment because the noise still lies within the desirable range for larvae, then acoustic enrichment may not be needed at such sites. We, therefore, suggest that in the planning stages of restorations that intend to use acoustic enrichment, the soundscapes of candidate sites be surveyed to establish whether acoustic enrichment might provide a sound-boosting effect relative to any anthropogenic noise. If noise is disruptive but can be mitigated [82], or if technological solutions in acoustic technology provide navigational cues that penetrate this noise, then acoustic enrichment might still yield positive ecological and economic returns on investment. Of note, the volume of our speakers in the field were confined to a small area to avoid sound crossover with the control sites, as also arranged at the natural site of low anthropogenic noise. The low setting of volume may have enabled the background noise at the anthropogenic site to mask the acoustic enrichment from the speaker more readily. We, therefore, suggest that future studies test for such nuances to determine the levels at which speakers might overpower anthropogenic noise, thereby increasing the effect sizes of the boosted sound level and larval recruitment. It seems useful, therefore, to understand the role of natural and anthropogenic soundscapes on recruitment processes to inform where, when and how we manage and restore marine ecosystems.

## 5. Conclusion

Reducing the bottlenecks to natural recruitment of target organisms represents a critical step towards achieving marine restoration success. In particular, the replenishment of reef-building populations is key to rebuilding the foundations and function of reef habitats. Innovative solutions like acoustic enrichment, when complemented with other restoration efforts (e.g. substrate provision), could enhance this key process of restoration, potentially reducing the risk of recruitment failure by ensuring a steady supply of recruits to seed recovery. We show that using acoustic enrichment at a relatively quiet, natural site can boost the recruitment of a key reef-building organism. However, our pioneering assessment provides new insight on how anthropogenic noise appears to mask acoustic enrichment, and may disrupt the recruitment of oyster larvae, highlighting the context dependency of using acoustic enrichment for reef restoration. We are aware that we have not been able to distinguish between the volume of noise from the type of noise that drives differences in recruitment and distinguishing between the two would represent a major contribution to understanding whether and how to manage noise pollution. If anthropogenic noise masks acoustic enrichment of healthy reef sounds and disrupts recruitment processes, the role of acoustic technology may be reduced for restoration efforts. These concerns may be appropriate where marine restoration efforts are focused along noisy, metropolitan coastlines and urbanized waterways. But where there is little anthropogenic noise, acoustic enrichment appears to enhance the process of recruitment which is key to restoration success.

## Data Availability

All data and sound files are publicly accessible through the data repository [83]. Supplementary material is available online [84].
